# Factors associated with knowledge and practice of breast self-examination among female governmental school teachers in Gondar Town, Northwest Ethiopia, 2019

**DOI:** 10.3389/fonc.2024.1481714

**Published:** 2024-12-24

**Authors:** Birhaneslasie Gebeyehu Yazew, Biresaw Wassihun Alemu, Tarkie Abebe Walle

**Affiliations:** ^1^ Department of Nursing, College of Medicine and Health Sciences, Injibara University, Injibara, Ethiopia; ^2^ Department of Midwifery, College of Medicine and Health Sciences, Injibara University, Injibara, Ethiopia; ^3^ Department of Surgical Nursing, School of Nursing, College of Medicine and Health Sciences, University of Gondar, Gondar, Ethiopia

**Keywords:** breast self-examination, knowledge, practice, female teachers, Ethiopia

## Abstract

**Background:**

Breast cancer is the most common cancer among women globally, and early detection through breast self-examination can improve survival rates. However, this practice is limited in developing countries like Ethiopia.

**Objective:**

This study aimed to assess the factors influencing knowledge and practices related to breast self-examination among female governmental school teachers in Gondar Town, Northwest Ethiopia.

**Methods:**

A cross-sectional study was conducted from May 1 to 30, 2019, with 422 female teachers in Gondar metropolis governmental schools selected through simple random sampling. Data were collected using a self-administered questionnaire and analyzed using EPI INFO version 7 and SPSS version 20.

**Result:**

A total of 415 female teachers participated in the study, with a response rate of 98.3%. The mean age of respondents was 38.64 years. Only 41.9% had good knowledge of breast self-examination, while 14.5% reported good practice. Factors associated with knowledge included secondary education, higher degrees, and exposure to information. Factors influencing practice included having a degree or higher education and prior experience with breast self-examination.

**Conclusion and recommendation:**

The study revealed low levels of knowledge and practice of breast self-examination among female teachers. Educational level was significantly associated with both knowledge and practice. Recommendations include implementing health education campaigns, organizing events like breast cancer awareness days, and forming support groups in schools to promote awareness and encourage regular breast self-examination among female teachers in Ethiopia.

## Introduction

Breast cancer (BC) is a prevalent form of cancer that primarily affects women (1). It is a significant public health concern, resulting in high levels of morbidity and mortality. Each year, over 2.1 million women are diagnosed with breast cancer worldwide, with more than half of them succumbing to the disease (2). The incidence of breast cancer is increasing in both developed and developing countries. In 2020, there were 2.3 million newly diagnosed cases and 685,000 deaths worldwide (3).

According to statistics, breast cancer accounts for approximately 25% of cases and 15% of deaths worldwide (4). By the end of 2020, it is projected that 43.1% of women globally and 36.8% in underdeveloped countries will succumb to breast cancer (5). This regional trend of increasing morbidity and mortality due to breast cancer is concerning, particularly in countries with limited resources, as a result of factors such as increased lifespan, urbanization, and adoption of Western lifestyles (6). Therefore, there is an urgent need for action to prevent and detect breast cancer early through various screening methods in order to achieve the sustainable development goal by 2030 (7). In contrast, the rise in cancer cases was observed in developing areas, accounting for 56% of new cancer cases and 63% of cancer-related deaths (8). In Ethiopia, breast cancer is the most prevalent type among adults, making up 30.2% of cases. The majority of reported annual cancer-related deaths in Ethiopia are women (9). Breast cancer is the second leading cause of death, responsible for 2.7% of all mortalities and ranking among the top five causes of non-communicable deaths. While efforts are being made to establish cancer centers in Ethiopia, these facilities are not as extensive as needed (10). Compared to those in Tanzania, Ethiopia’s service centers for cancer care are more advanced but fall short of those in Zambia (11). Many healthcare facilities in Ethiopia lack the necessary modern laboratory equipment for breast cancer screening and diagnosis.

Therefore, early detection programs such as breast self-examination, objective breast health checkups, and mammography can help minimize the morbidity and mortality associated with breast cancer (12). In Ethiopia, a National Cancer Control Plan was established in 2015 (9), with breast self-examination (BSE) being promoted as a crucial method for early detection to improve survival rates (13). The key components of BSE involve pictorial checkups and palpation of the breast, which have been shown to empower women to take control of their breast health (14, 15). Additionally, BSE is a valuable screening tool, with nursing and teaching professions playing a significant role in raising community awareness through education (16). Increased awareness of BSE among female teachers can directly or indirectly improve the resistance and quality of life for breast cancer patients (17) and themselves by enabling early detection and treatment initiation (18, 19).

Various studies have found varying levels of knowledge and performance of breast self-examination among women, despite its importance. For example, research in Iraq found that only 15.7% had good knowledge of BSE (20), while in Sudan, the number was 34% (21); in Cameroon, it was 78% (22); in different regions of Ethiopia (specifically Arba Minch, Jima, and Adwa Town), the percentages ranged from 34.2% to 55.5% (23–25).

According to the practices of women in various countries, the percentage of study participants implementing BSE was as follows: University Putra Malaysia 36.7% (26), Pakistan 24.9% (27), Ghana 37.6% (28), Cameroon 38.5% (29), Egypt 39.2% (30), and Sudan 20.6% (21). In Addis Ababa, Ethiopia, the percentages were 18.6% and 13.1% (31, 32).

Different researchers have identified various factors that may influence the knowledge and practice of BSE among women, including age, income, educational level, the educational and professional status of the husband, and source of information (32–35). However, these findings are inconclusive.

In developing countries where diseases are often diagnosed late due to limited resources, early detection of cases through breast self-examination is crucial. The practice of BSE has been shown to empower women to take control of their health (5). Female teachers who are knowledgeable about and perform BSE can have a positive impact on mothers, students, and other women in the community by sharing information and serving as role models. However, there is limited evidence in the specific study area. Therefore, this study aims to evaluate the knowledge, practices, and factors associated with breast self-examination among governmental school teachers in Gondar Town, Northwest Ethiopia.

## Materials and methods

### Sample and setting

A facility-based cross-sectional study was conducted in Gondar Town, Northwest Ethiopia, from May 1 to 30, 2019. Gondar Town, located 734 km from the capital city of Addis Ababa, is an ancient and densely populated town in Ethiopia. The town has experienced growth and currently has eight health centers and one referral hospital serving the population and surrounding areas. According to the 2019 report from the central Gondar zonal educational office, there are a total of 81 schools in Gondar Town, with 55 being governmental and 26 non-governmental. Among the teachers, 1,596 are female, with 1,229 in governmental schools and 367 in non-governmental schools.

The study population consisted of all female teachers working at governmental schools in Gondar Town, Northwest Ethiopia, during the data collection period. Only female teachers between the ages of 20 and 70 who were not undergoing treatment for mastectomy or were seriously ill were included in the study.

The sample size was determined using the single population proportion formula by $n=\frac{{\left(\frac{Za}{2}\right)}^{2}\;P\left(1-P\right)}{d^{2}}$ , considering the 50% assumption of 95% confidence interval, 5% margin of error, and 10% possible non-response rate. Then, the final sample size was 422.

The sample was acquired by distributing the sample size proportionally among each level of school (574 for elementary, 396 for secondary, 206 for high school, and 53 for preparatory). Each study participant was then selected using a simple random sampling technique with the lottery method, based on the female teachers’ roster from the Gondar Town educational office, from each school (179 from elementary, 136 from secondary, 71 from high school, and 18 from preparatory). Ultimately, 422 female teachers were chosen. Elementary schools include first to fourth grades, secondary schools encompass fifth to eighth grades, high school covers ninth and 10th grades, and preparatory schools involve 11th and 12th grades.

### Operational definitions

In this study, good knowledge of BSE was defined as those who answered more than 60% of the knowledge questions correctly, with those scoring 60% or below considered to have poor knowledge. Similarly, good practice was defined as scoring more than 60% on the practice-related questions, while those scoring 60% or lower were considered to have poor practice (36).

### Data collection tool and data analysis

We utilized a standardized structured self-administered questionnaire adapted from relevant literature for our study (27, 36). The questionnaire comprised four sections covering socio-demographic factors, sources of information and history of breast cancer-related factors, knowledge of BSE, and practice of BSE. To maintain data integrity, efforts were made from developing the data gathering tools to thoroughly checking the completed questionnaires. Necessary modifications were made based on the results of a pretest, which involved 5% of female teachers from Dessie Town. The reliability of the questionnaire was assessed using Cronbach’s alpha, with knowledge scoring 0.82 and practice scoring 0.74. Data collectors and supervisors received 1 day of training on the instruments to ensure quality data collection.

Before analysis, the gathered information was reviewed for completeness, accuracy, and clarity. The data were then entered into EPI INFO version 7.2 and analyzed using SPSS version 20. Descriptive statistics, such as frequencies and proportions, were used to summarize the study variables. Binary logistic regression models were employed to identify associated factors, with variables having a p-value ≤0.2 in the bivariate analysis being included in the multivariate analysis to control for confounding factors. Odds ratios with 95% confidence intervals were calculated to determine the strength of the associations. A significance level of p < 0.05 was considered statistically significant.

## Result

### Socio-demographic characteristics of the study participants

A total of 415 individuals participated in the study, resulting in a response rate of 98.3%. Within the age category of 33–43 years, 145 participants were identified, accounting for 34.9% of the total. The majority of respondents, 285 individuals (68.7%), identified as Orthodox Christians. In terms of salary/income, 68% of participants earned more than or equal to 4,000.00 ETB, while 32% earned less than 4,000.00 ETB. The distribution of participants according to department level was as follows: 26.7% social, 29.6% cluster, and 43.6% natural (**Supplementary Table S1**).

### The source of information about BSE and history of breast cancer

The majority, 401 (96.6%), of the study participants did not have previous breast cancer or any cancer, and only 35 (8.4%) of their families had a history of breast cancer. Of the study participants, 297 (71.6%) respondents have heard information about breast self-examination. Of those who had information about BSE, 164 (55.2%) of them were heard from healthcare workers and 59 (19.9%) from friends/peer groups, and only 74 (24.9%) were from newspapers, magazines, and the Internet.

### Knowledge of school teachers towards BSE

More than half of the study participants, 243 (58.6%), recognized the importance of BSE in the early detection of breast cancer. Of the participants, 172 (41.2%) correctly stated that BSE should be performed monthly. However, 311 (74.9%) of participants inaccurately believed that a history of a breast lump does not increase the risk of breast cancer, and 197 (47.5%) thought that examining the armpit during BSE is necessary for detecting lumps. Only 81 (19.5%) of participants knew that both men and women can perform BSE. The majority of participants, 328 (79%), believed that BSE should be conducted by health professionals.

### The practice of school teachers of BSE

Regarding questions related to practice, 167 (40.2%) of participants in the study indicated that breast self-examination should be conducted when it is thought of, while 164 (39.5%) correctly stated that palpation with the palm and three fingers is the appropriate method for conducting breast self-examination. In addition, 189 (45.5%) of participants believed that breast self-examination should start at the age of 40 and above, and 237 (57.1%) thought that examining only the left side of the breast was sufficient during the process. Only 37.0% of participants had ever performed breast self-examination, with 29.6% of those who had not performed stating that they did not know how to perform it. Fear of detecting an abnormality was the reason given by only 2.4% of participants for not conducting breast self-examination. Overall, the study found that 14.5% of participants demonstrated good practice in performing breast self-examination, scoring between 1 and 7 on the practice assessment scale (**Supplementary Table S2**).

### Factors associated with knowledge of BSE

In the bivariate logistic regression model, all independent variables were included, and those with a p-value ≤0.2 were further analyzed in a multivariate analysis to control for confounding factors. In the multivariate analysis, three variables showed a significant association with the knowledge of female school teachers on BSE. Female school teachers with qualifications of a Bachelor of Science (BSc) or higher were approximately six times more likely to be knowledgeable about BSE compared to those with certificates and diplomas [adjusted odds ratio (AOR): 5.5, 95% CI: 1.65–18.4]. Additionally, secondary school (grades 5–8) female teachers were approximately three times more likely to have knowledge of BSE compared to primary school teachers (AOR: 2.6, 95% CI: 1.12–8.87). Female school teachers who had exposure to information were nearly five times more likely to be knowledgeable about BSE compared to those without exposure (AOR: 4.9, 95% CI: 1.71–4.63) (**Table 1**).

**Table 1:** Bivariable and multivariate logistic regression analysis for factors that affect knowledge of female school teachers on BSE, Gondar town, Northwest Ethiopia, 2019 (n = 415).

Variables	Categories	Knowledge		COR (95% CI)	AOR (95% CI)	p-Value
		Good	Poor			
Religion	Orthodox Muslim Protestant	111 50 13	174 38 29	1 2.06 (0.8, 5.2) 1.42 (0.19, 4.82)	1 1.4 (0.42, 3.81) 0.72 (0.46, 2.33)	0.37 0.06
Level of education	Diploma and certificate	65	187	1	1	
	Degree and above	109	54	5.8 (2.42, 10.7)	5.5 (1.65, 18.4)	0.02*
Level of school	Primary school (grades 1–4)	44	134	1	1	
	Secondary school	28	44	1.94 (1.3, 8.29)	2.6 (1.12, 8.87)	0.04*
	High school (grades 9 and 10)	16	15	3.25 (1.49, 4.13)	4.74 (1.6, 8.7)	0.001*
	Preparatory school	86	48	5.46 (1.79, 14.3)	4.82 (3.71, 9.92)	0.03*
Exposure to information about BSE	No	25	93	1	1	
	Yes	168	129	4.84 (4.62, 6.2)	4.9 (1.703, 4.63)	0.001*

p is statistically significant at p < 0.05 level of significance.

BSE, breast self-examination; COR, crude odds ratio; AOR, adjusted odds ratio.

*Statistically significant.

### Factors associated with the practice of breast self-examination

In the multivariate analysis, two variables were found to be independently associated with the practice of BSE among female school teachers. According to the results, female school teachers with a degree or master’s degree were approximately three times more likely to have good practice compared to those holding certificates or diplomas (AOR: 2.94, 95% CI: 1.01–8.58). Additionally, female teachers who had previously performed BSE were two times more likely to have good practice compared to those who had not performed BSE (AOR: 2.38, 95% CI: 1.04–5.41) (**Table 2**).

**Table 2:** Bivariable and multivariate logistic regression analysis for factors that affect the practice of female school teachers on BSE, Gondar town, Northwest Ethiopia, 2019 (n = 415).

Variables	Categories	Practice		COR (95% CI)	AOR (95% CI)	p-Value
		Good	Poor			
Level of education	Certificate and diploma	12	240	1	1	
	Degree and above	49	114	8.6 (1.6, 6.2)	2.94 (1.01, 8.58)	0.001*
Level of school	Primary Secondary High school Preparatory	16 23 15 7	162 49 16 127	1 4.75 (0.6, 17.51) 9.5 (4.73, 14.45) 0.56 (0.17, 6.52)	1 2.3 (0.25, 16.2) 2.6 (0.79, 3.68) 4.57 (0.71, 7.01)	0.28 0.06 0.67
History of breast cancer	No Yes	55 6	346 8	1 4.71 (0.85, 7.01)	5.95 (0.59, 8.91)	0.45
Family history of breast cancer	No Yes	52 9	328 26	1 2.18 (0.63, 12)	2.43 (0.27, 14.67)	0.62
Exposure to information about BSE	No	8	110	1	1	
	Yes	53	244	2.98 (1.14, 6.12)	2.38 (1.046, 5.41)	0.02*
Knowledge	Poor Good	22 39	219 135	1 2.87 (0.6, 2.07)	2.86 (0.85, 3.42)	0.07

p is statistically significant at p ≤ 0.05 level of significance.

BSE, breast self-examination; COR, crude odds ratio; AOR, adjusted odds ratio.

*Statistically significant.

## Discussion

The World Health Organization (WHO) is stressing the importance of including cancer prevention efforts in overall cancer control strategies, as nearly 40% of cancer deaths are currently being overlooked (37). BSE is considered a key part of these strategies, and this research aimed to investigate the factors influencing the knowledge and practice of BSE among female school teachers in Gondar Town. The study found that 41.9% of governmental school teachers had good knowledge of BSE, a similar percentage to a study conducted in Addis Ababa, Ethiopia (38). The similarity may be due to alike socio-cultural and economic status. However, this finding was lower than that of the studies conducted in other countries: Pakistan 71.4% (27), India (39), Jordan 93.1% (40), Sub-Saharan countries 81.5% (41), Nigeria 54% (42), and Cameroon 78% (22). The possible justification may be due to study setting difference (22), the difference in sampling method (non-probability in Sub-Saharan African study) (41), the study population, with some of the above studies conducted among health students (41), and a surgical outpatient (22). Furthermore, it may be due to the difference in socioeconomics, access to sources of information (Internet and media access), and healthcare services (27, 39, 40).

In contrast, the findings of this study were higher than those of the studies conducted in Arab American University (43), Iraq 15.7% (20), Sudan 34% (21), and different regions of Ethiopia (Debre Berhan 30.25% and Adama 8.7%) (44, 45). The possible justification for these discrepancies may be the study settings (21, 43), the student population (44), the small sample size (210) (20), and the method of analysis they used (ordinal logistic regression) at Adama, Ethiopia (45).

The other outcome variable of this study was the implementation or practice of BSE. Then, the good practice of BSE among female teachers was 14.5% (95% CI: 10.6%–19.1%). This finding was similar to that of the studies conducted in Malaysia 19% (46) and Sub-Saharan Africa (41). This may be due to a similar study population (the Malaysian study was conducted among female teachers) and Sub-Saharan African countries having similar health-seeking behavior.

However, the finding of this study on good practice of BSE was lower than that of the studies conducted in other countries: Malaysia 36.7% (26), Pakistan 24.9% (27), India 33.3% (47), Ghana 37.6% (28), Cameroon 38.5% (29), Egypt 39.2% (30), Sudan 20.6% (21), Zambia 28.2% (48), Nigeria 38% (42), and different regions of Ethiopia (Jimma 21%, Adama 39.4%, and Addis Ababa 21.4%) (36, 45, 49). The reason for the differences in findings between this study and others may be attributed to variations in study settings (26–28, 30, 42, 47, 48) and access to healthcare (50). Other studies may have included populations with different characteristics, such as students enrolled in health-related courses or excluding individuals under the age of 21, which could impact their ability to practice and promote breast self-examination (21, 29, 36, 45, 49). Conversely, the results of this study showed a higher percentage of participants practicing BSE compared to studies conducted in India (4.5%) (39) and Adwa, Ethiopia (6.25%) (25). These variations could be due to differences in study settings, tools used, cutoff points for defining good practice, and the educational levels of the study populations.

Regarding factors associated with good knowledge of BSE in this study, the level of education of study participants was positively associated with knowledge of BSE. Thus, those with degrees (BSc) and above were approximately six times more likely to be knowledgeable about BSE than certificate and diploma holders. This was supported by the study conducted in Adama, Ethiopia, which reported that a statistically significant association was obtained between knowledge of BSE and the level of education of the participant (45). This may be due to the fact that if someone’s educational status increases, his/her knowledge status is also concomitantly enhanced (51).

In terms of factors associated with good knowledge of BSE in this study, participants with higher levels of education (BSc degrees and above) were significantly more knowledgeable about BSE compared to those with certificates or diplomas. This finding is consistent with a study conducted in Adama, Ethiopia (45), which also found a significant association between level of education and knowledge of BSE (52). This suggests that as individuals’ educational levels increase, so does their knowledge and understanding of BSE. Participants who had knowledge of breast self-examination were more likely to have a good understanding of BSE, similar to research conducted in Malaysia (53). This could be attributed to increased awareness leading to participation in campaigns and following health personnel guidance on BSE.

The study also found a positive correlation between educational level and practicing breast self-examination. Women with a degree or master’s degree were three times more likely to have good practice compared to those with certificates or diplomas, in line with findings from studies in Adama, Ethiopia (45), and Nigeria (54). Higher educational levels may enable women to access information and develop effective techniques for performing BSE to prevent diseases (55).

In addition, regularly performing BSE was linked to better practice of breast self-examination. Female teachers who had previously conducted BSE were twice as likely to have good practice compared to those who had not. This is consistent with previous research (56, 57) suggesting that a lack of prior BSE performance may result in lower competence in practicing the technique. A history of performing BSE can positively influence the ability to conduct breast examinations effectively.

## Conclusion

This research indicated a lack of knowledge and limited practice of breast self-examination among female school teachers. The study also found that educational level, receiving information about BSE, and school level positively correlated with better knowledge of breast self-examination. Additionally, educational level and practicing BSE correlated positively with better performance on the examination. Therefore, it is crucial to implement health education campaigns, observe events like breast cancer awareness day, and establish groups within schools to promote awareness and encourage regular practice of breast self-examination among female teachers.

## Strength and limitation

This study may be subjected to response bias, and it may be underreported or over-reported. Furthermore, it focused only female teachers only.

## Data Availability

Requests to access these datasets should be directed to BY, Kassish6@gmail.com.
