# Supplementary material for: Factors associated with knowledge and practice of breast self-examination among female governmental school teachers in Gondar Town, Northwest Ethiopia, 2019
Source: Front Oncol. 2024 Dec 24;14:1481714. doi: 10.3389/fonc.2024.1481714 (PMC11703965; doi:10.3389/fonc.2024.1481714)
Supplement: Supplementary file 1 [file DataSheet1.docx]

**Table s1: Socio-demographic characteristics of the female teachers at governmental school, Gondar town, Northwest Ethiopia, 2019; (n=415)**

| **Questions** | **Categories** | **Frequency** | **Percent** |
| --- | --- | --- | --- |
|  |  |  |  |
|  |  |  |  |
| **Age of the study participants** | 22-32 years | 143 | 34.5 |
|  | 33-43 years | 145 | 34.9 |
|  | ≥44 years | 127 | 30.6 |
|  |  |  |  |
| **Religion** | Orthodox | 285 | 68.7 |
|  | Muslim | 88 | 21.2 |
|  | protestant | 40 | 9.6 |
|  | *others | 2 | .5 |
| **Marital Status** | single | 65 | 15.7 |
|  | married | 282 | 68.0 |
|  | widowed | 44 | 10.6 |
|  | Divorced | 24 | 5.8 |
| **level of education** | certificate | 9 | 2.2 |
|  | Diploma | 243 | 58.6 |
|  | Degree | 151 | 36.4 |
|  | Master | 12 | 2.9 |
| **level of school** | secondary school (5-8th grade) | 144 | 34.7 |
|  | high School (9 & 10 grade) | 174 | 41.9 |
|  | Preparatory school(11 & 12 grade) | 97 | 23.4 |

* Others=Catholic

**Table s2: Practice of school teachers about BSE, Gondar Town, Northwest Ethiopia, 2019; n=415**

| **Questions** | **Categories** | | **Frequency** | **Percent** | | |
| --- | --- | --- | --- | --- | --- | --- |
| **When to be performed Breast self- examination?** | a week after menses* | | 122 | 29.4 | | |
|  | When it comes to mind | | 167 | 40.2 | | |
|  | any time during menses | | 80 | 19.3 | | |
|  | a week before menses | | 46 | 11.1 | | |
| **How is breast self-examination done** | Palpate with one finger | | 94 | 22.7 | | |
|  | palpate with palm and three fingers* | | 164 | 39.5 | | |
|  | I don't know | | 157 | 37.8 | | |
| **At what age did you start Breast self-examination** | Before 20 years | | 38 | 9.2 | | |
|  | **>**20 years* | | 188 | 45.3 | | |
|  | 40 years and above | | 189 | 45.5 | | |
| **What time do you normally perform Breast self-examination be done?** | Morning* | | 196 | 47.2 | | |
|  | afternoon | | 104 | 25.1 | | |
|  | Evening | | 115 | 27.7 | | |
| **Where do you usually perform Breast self-examination** | In front of mirror* | | 101 | 24.3 | | |
|  | Everywhere | | 233 | 56.1 | | |
|  | I do not know | | 81 | 19.5 | | |
| **What do you do if you feel pain** | Tell mother/mother-in-law | | 89 | 21.4 | | |
|  | Tell spouse | | 86 | 20.7 | | |
|  | Consult doctor/nurse* | | 181 | 43.6 | | |
|  | Not do anything about it due to embarrassment | | 36 | 8.7 | | |
|  | Go to traditional healing | | 23 | 5.5 | | |
| **During Breast self-examination, it is enough to see left side of the breast only** |  |  |  |  |  |  |
|  | No* | | 178 | 42.9 | |  |
|  | Yes | | 237 | 57.1 | |  |
| Table s2: Practice of school teachers about Breast self-examination, Gondar Town, Northwest Ethiopia, 2019 con… | | | | | |  |
| **Breast self-examination can always be done by health professionals** | |  |  |  |  |  |
|  |  | Yes | 137 | 33.0 |  |  |
|  |  | No* | 278 | 67.0 |  |  |
|  | |  |  |  |  |  |

* Correct answer.


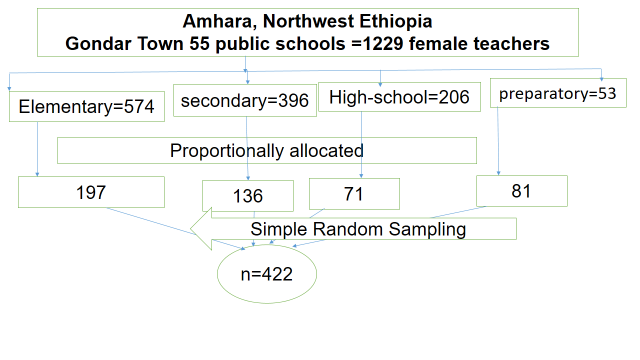


**Figure s1:** Schematic presentation of participants sampling technique at Gondar Town governmental Schools, Northwest Ethiopia, 2019
